# A NEW approach for characterizing mouse urinary pathophysiologies

**DOI:** 10.14814/phy2.14964

**Published:** 2021-08-02

**Authors:** Hannah M. Ruetten, Gervaise H. Henry, Teresa T. Liu, Heidi M. Spratt, William A. Ricke, Douglas W. Strand, Chad M. Vezina

**Affiliations:** ^1^ Department of Comparative Biosciences University of Wisconsin‐Madison Madison WI USA; ^2^ University of Wisconsin‐Madison/UMASS Boston/UT Southwestern George M. O’Brien Center for Benign Urologic Research Madison WI USA; ^3^ Department of Urology UT Southwestern Medical Center Dallas TX USA; ^4^ Department of Urology University of Wisconsin‐Madison Madison WI USA; ^5^ Department of Preventive Medicine and Population Health University of Texas Medical Branch Galveston TX USA

**Keywords:** animal models, bladder outlet obstruction, diabetic diuresis, spontaneous void spot assay, urinary frequency

## Abstract

The void spot assay (VSA) is a cost‐effective method for evaluating and quantifying mouse urinary voiding phenotypes. The VSA has been used to differentiate voiding behaviors between experimental groups, but not as a diagnostic assay. To build toward this goal, we used the VSA to define voiding patterns of male mice with diabetic diuresis (BTBR.Cg‐*Lep* ^ob^ /WiscJ mice), irritative urinary dysfunction (*E*. *coli* UTI89 urinary tract infection), and obstructive urinary dysfunction (testosterone and estradiol slow‐release implants) compared to their respective controls. Many studies compare individual VSA endpoints (urine spot size, quantity, or distribution) between experimental groups. Here, we consider all endpoints collectively to establish VSA phenomes of mice with three different etiologies of voiding dysfunction. We created an approach called normalized endpoint work through (NEW) to normalize VSA outputs to control mice, and then applied principal components analysis and hierarchical clustering to 12 equally weighted, normalized, scaled, and zero‐centered VSA outcomes collected from each mouse (the VSA phenome). This approach accurately classifies mice based on voiding dysfunction etiology. We used principal components analysis and hierarchical clustering to show that some aged mice (>24 m old) develop an obstructive or a diabetic diuresis VSA phenotype while others develop a unique phenotype that does not cluster with that of diabetic, infected, or obstructed mice. These findings support use of the VSA to identify specific urinary phenotypes in mice and the continued use of aged mice as they develop urinary dysfunction representative of the various etiologies of LUTS in men.

## Graphical abstract

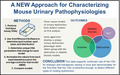

## 1 INTRODUCTION

Urine voiding and storage symptoms (collectively known as lower urinary tract symptoms, or LUTS) are common in aging men, decrease quality of life, and often coexist with a progressive disease that can include recurrent urinary tract infections, bladder caliculi, bladder wall remodeling, acute urinary retention, and renal impairment (Irwin et al., 2011; Kellogg Parsons et al., 2008). Patient‐based surveys such as the American Urologic Association‐Symptom Index (AUA‐SI), International Prostate Symptom Score (IPSS), and NIH‐Chronic Prostatitis Symptom Index (NIH‐CPSI) are used to quantify human LUTS for medical and clinical research purposes (Barry et al., 1992; Litwin et al., 1999). Survey outcomes inform selection of diagnostics or therapeutics and are used to monitor disease progression and evaluate treatment outcomes (Barry et al., 1992; Litwin et al., 1999).

The widespread use of patient‐based survey data for human LUTS creates challenges for modeling urinary dysfunction in non‐human species such as mice, in which surveys are neither practical nor possible. Alternative approaches focus on urinary physiology and include cystometry, uroflowmetry, contrast enhanced ultrasound, and others. These methods can identify voiding function differences between experimental groups (Bjorling et al., 2015; Keil et al., 2016; Liu et al., 2019; Ruetten et al., 2019, 2021; Turco et al., 2020; Wegner et al., 2018, 2020) but are not proven diagnostic assays. For example, it is currently not possible to apply, in a blinded fashion, a single voiding function assessment to a mouse and use the experimental outcomes to accurately predict the underlying disease etiology (e.g., diabetic diuresis, irritative voiding, obstructive voiding, neurogenic voiding, or other).

We use the spontaneous void spot assay (VSA) as one component of a comprehensive mouse voiding function assessment. The assay involves placement of mice on an absorbent filter paper for a fixed time interval, then visualization and quantification of void spots on the filter paper. A free software program enables objective, consistent, and rapid analysis of VSA filter paper images to generate summative VSA endpoint data such as total void spot number, average void spot size, distribution of spots in the corners and centers of the paper (Wegner et al., 2018). Because the assay has been standardized and is reproducible across laboratories (Hill et al., 2018), our long‐term goal is to build the VSA into a rapid and inexpensive diagnostic assay for mice.

In previous applications, single VSA endpoints such as void spot number were compared between experimental groups. Here, we considered twelve endpoints, generated as part of the standard output from the VSA analysis software Void Whizzard (Wegner et al., 2018), as equally weighted variables to establish a collective VSA phenome for each mouse. The purpose of this study was to test whether mice harboring distinct etiologies of voiding dysfunction could be accurately classified based solely on VSA phenome. We applied an approach that we named normalized endpoint work through (NEW) and used principal components analysis (PCA) and hierarchical clustering to show that the VSA phenome of 6‐ to 10‐week‐old male mice with diabetic diuresis (driving by a leptin mutation, *Lep^ob^* ^/^ *^ob^*, in BTBR mice), irritative voiding from UTI (intraurethral *E*. *coli* UTI89) and bladder outlet obstruction (driving by slow‐release, subcutaneous testosterone, and estradiol implants) are fairly unique. We also show that aged mice (>24 m old) do not uniquely cluster as a single group based on VSA phenome. Instead, some aged mice cluster with diabetic mice, other aged mice cluster with urinary tract infected mice, and others cluster independently, suggesting that aging generates a spectrum of spontaneous voiding disorders in mice, as in humans.

## 2 MATERIALS AND METHODS

### 2.1 Void spot assay and mice

This study uses VSA data from previously published studies (Liu et al., 2019; Ruetten et al., 2021; Wegner et al., 2018, 2020). In all studies, experiments were conducted under approved Animal Care and Use Committee Protocols at the University of Wisconsin‐Madison. All VSAs were conducted according to best practices published by Hill *et al*. and as described in the Void Whizzard user manual (Hill et al., 2018; Wegner et al., 2018). VSAs were performed in the same vivarium where mice were housed, and mice had access to food but not water during the 4‐hour evaluation period. VSAs were conducted on experimental mice and their respective controls at the same time of day and using Whatman grade 540 (Fisher Scientific #057163W) filter papers (27 x 16 cm) secured with masking tape to the cage bottom. Filter papers were imaged under ultraviolet light and the resulting 16‐bit images were processed by Void Whizzard (Wegner et al., 2018) to generate a summative VSA endpoint dataset which includes: total spot count, total void area (cm^2^), % area in center of paper, % area in corners of paper, and mass distribution of spots (0–0.1, 0.1–0.25, 0.25–0.5, 0.5–1, 1–2, 2–3, 3–4, 4+ cm^2^). All standard variables included in the Void Whizzard output were used without alteration, including standard units for spot size (cm^2^), and default binning of spot sizes. A summary of mice and experimental variables for this study is listed in Table 1, and a comprehensive description of the procedures for blood glucose testing, *E*. *coli* UTI89 infection and urine culture, and subcutaneous slow‐release testosterone and 17‐β‐estradiol implantation are described elsewhere (Liu et al., 2019; Ruetten et al., 2021; Wegner et al., 2018, 2020).

**TABLE 1:** Summary of mice used in this study

Name	Genetic Background		Jackson Labs #	Age	Treatment	Number of mice per group	Reference from which VSA endpoints were obtained
*Lep^Ob/Ob^*	BTBR		004824	8–10 wks	None	7	Wegner et al., 2018)
*Lep^+/+^*	BTBR		004824	8–10 wks	None	7	
						
UTI	C57BL/6J		000664	8 wks	Transurethral instillation of 100 µl of uropathogenic *E*. *coli* UTI89 in saline at an optical density of 0.70. Mice with <10,000 CFUs/ml free catch urine at 24 h were excluded.	6	Ruetten et al., 2021)
Control	C57BL/6J		000664	8 wks	Transurethral of 100 µl of saline	12	
T+E2	C57BL/6J		000664	6 wks	Subcutaneous compressed pellets of 25 mg testosterone and 2.5 mg 17‐β estradiol	10	Wegner et al.,)
Control	C57BL/6J		000664	6 wks	Sham surgery	10	
Old	C57BL/6J		000664	96 wks	None	5	Liu et al., 2019)
Young		C57BL/6J	000664	8 wks	None	5	

A previous study reported that some male mice exhibit a dominant/frequent voiding pattern (defined as a mouse that deposits >100 spots 0–0.1cm^2^ in size during a 4‐hour period)(Keil et al., 2016); these mice are sometimes excluded from the study and other times included. No mice were excluded from this study based on dominant/frequent voiding patterns. In the diabetic diuresis group, there were no dominant/frequent voiders. In the obstructive group, mice were singly housed for the study duration and had VSA performed 1‐week prior to study initiation to identify dominant/frequent voiders; there were no dominant/frequent voiders at initiation of the study. Two of ten control animals and six of ten T+E2‐treated animals developed a frequent spotter phenotype at the end of the study. In the irritative group, VSAs were performed at 24 h, 3 days, and 5 days post‐study initiation, no dominant/frequent voiders were identified at any of the early timepoints. At study endpoint, one of twelve control animals had a dominant/frequent voider phenotype.

This study utilizes data from previously published studies from which a sample size of 6–10 mice per group was sufficient to detect differences between control and treated animals. The present study involves groupwise and not pairwise comparisons, and group sizes are limited by the original study designs.

### 2.2 Statistical analyses

Statistical analyses were performed with Graph Pad Prism 8.0.2 to identify VSA endpoint differences between each experimental group and their respective control. Differences were considered significant at the *p* < 0.05 level. A Shapiro–Wilk test was used to test for normality and transformation was applied to normalize data. Bartlett's test was used to test for homogeneity of variance. The Student's *t* test was applied when variances were equal and a *t* test with Welsh's correction was applied when variance were unequal between groups. A Mann–Whitney test was performed when data could not be normalized through transformation.

**FIGURE 1:**
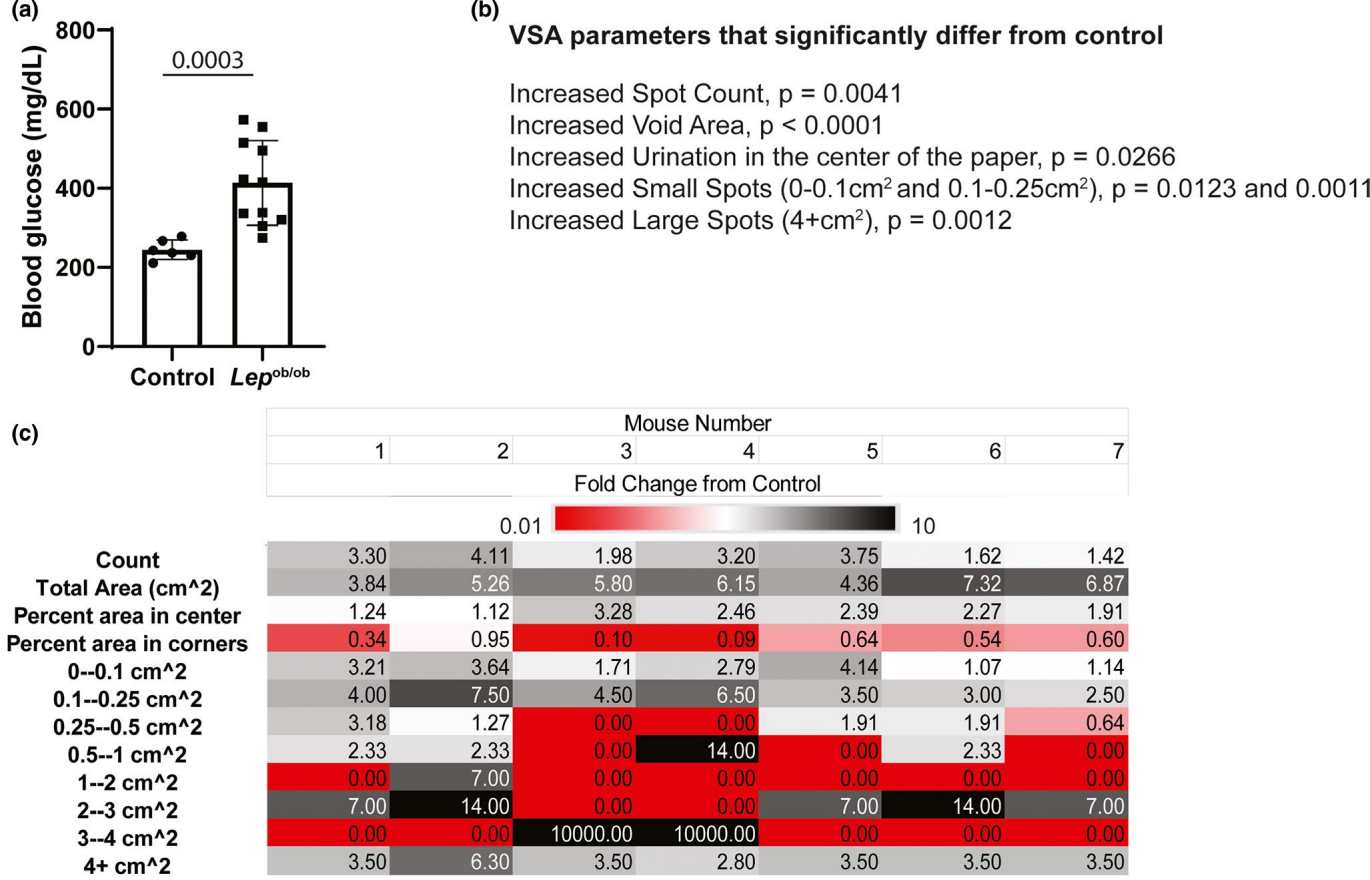
BTBR. Cg‐*Lep* ^ob^ /WiscJ mice develop hyperglycemia and urinary dysfunction consistent with polyuria. 8–10‐week‐old obese diabetic BTBR *Lep^ob^* ^/^ *^ob^* mice and wild type littermates (controls) were evaluated. (a) Tail vein blood was obtained to measure blood glucose concentrations after 4 h of fasting. Groups were compared using Student's *t* test. *p* < 0.05 was considered significant. (b) VSA parameters were compared between BTBR *Lep^ob^* ^/^ *^ob^* mice and controls, included is a list of significantly changed parameters. Void spot assays were performed in the vivarium where mice were housed for 4 h. Void Whizzard software was used to obtain void count, total void area, % voids in center of the paper, % voids in the corners of the paper, and counts of various sized spots. Groups were compared for each parameter using Student's *t* test. *p* < 0.05 was considered significant. (c) The average control value of 7 mice was determined for each VWO parameter and then fold‐change determined for each mouse (1–7) in the experimental group (*fold change* = [*VSA endpoint value for each individual experimental mouse*]/[*average VSA endpoint value for control group*]) to evaluate consistency of the model. Fold‐changes were color coded using a 3‐color scale: red ≤0.01, white =0, black ≥10

**FIGURE 2:**
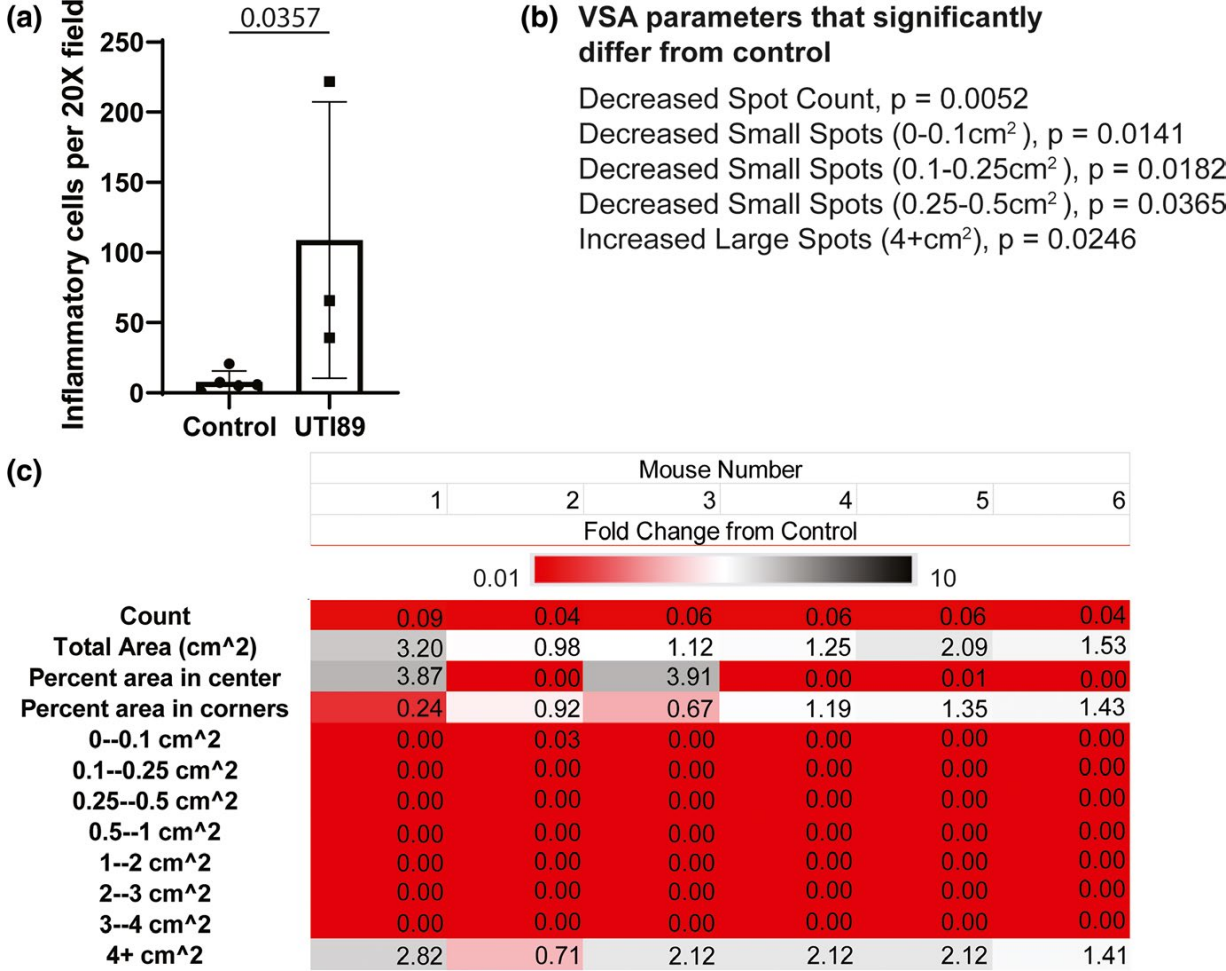
*E*. *coli* UTI89 infected mice develop prostatic inflammation and urinary dysfunction. A catheter was placed in anesthetized male mice (8 weeks) and mice were instilled with 100 µl sterile saline (control) or saline containing *E*. *coli* UTI89 at an optical density of 0.70. Mice with <10,000 colony forming units of *E*. *coli* UTI89 in free catch urine at 24 h were removed, leaving a population of 6 mice with a high level of infection and high probability of prostatic inflammation to model irritative urinary dysfunction. (a) Immunohistochemistry was performed for CD45 and positive cells counted per 20× field (435.84 µm × 331.42 µm) to determine number of inflammatory cells present. Cell counts from each group were compared using Student's *t* test. *p* < 0.05 was considered significant. (b) VSA parameters were compared between 6 *E*. *coli* UTI89 infected mice and 12 controls, included is a list of significantly changed parameters. Groups were compared for each parameter using Student's *t* test. *p* < 0.05 was considered significant. (c) The average control value was determined for each VWO parameter and then fold‐change determined for each mouse (1–6) in the experimental group (*fold change* = [*VSA endpoint value for each individual experimental mouse*]/[*average VSA endpoint value for control group*]) to evaluate consistency of the model. Fold‐changes were color coded using a 3‐color scale: red ≤ 0.01, white = 0, black ≥ 10.

**FIGURE 3:**
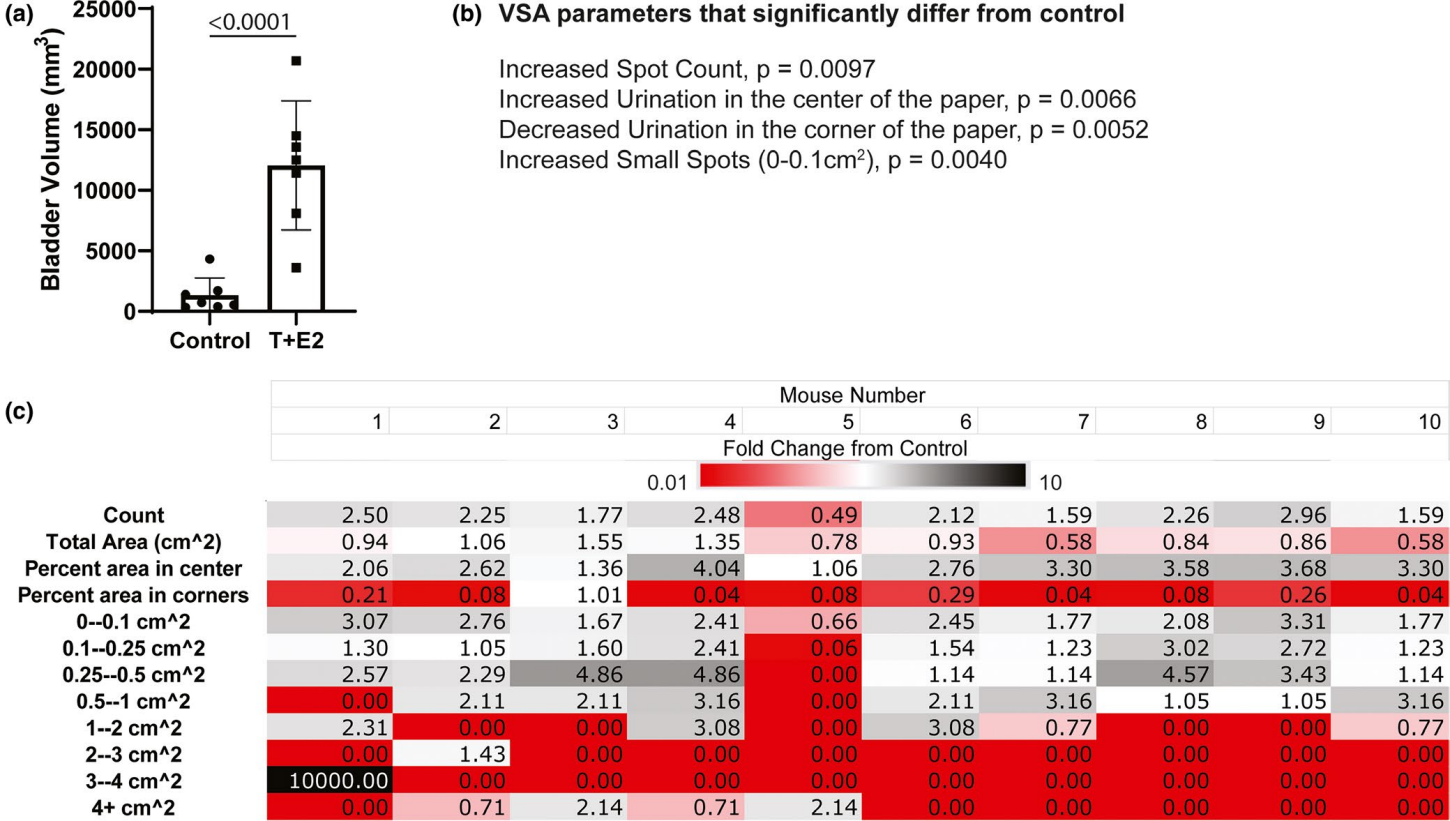
Testosterone and Estradiol (T + E2) supplemented mice develop distended bladders and urinary dysfunction. 6‐week‐old C57BL/6J mice were implanted with subcutaneous hormone implants (25 mg testosterone and 2.5 mg 17‐β estradiol) or a sham surgery (controls). (a) Bladder volumes were measured postmortem but prior to manually expressing the bladder to remove urine or to lower urinary tract removal. Groups were compared using Student's *t* test. *p* < 0.05 was considered significant. (b) VSA parameters were compared between T + E2 supplemented mice and controls, included is a list of significantly changed parameters. Void spot assays were performed for 4 h in the vivarium where mice were housed 4 weeks after hormone implant/ sham surgery. Void Whizzard software was used to obtain void count, total void area, % voids in center of the paper, % voids in the corners of the paper, and counts of various sized spots. Groups were compared for each parameter using Student's *t* test. *p* < 0.05 was considered significant. (c) The average control value was determined for each VWO parameter and then fold‐change determined for each mouse (1–10) in the experimental group (*fold change* = [*VSA endpoint value for each individual experimental mouse*]/[*average VSA endpoint value for control group*]) to evaluate consistency of the model. Fold‐changes were color coded using a 3‐color scale: red ≤ 0.01, white = 0, black ≥ 10

NEW was used to determine the fold‐change in VSA endpoints between experimental groups and their age‐, strain‐, and surgery‐matched controls using the following formula:

(*fold change* = [*VSA endpoint value for each individual experimental mouse*]/[*average VSA endpoint value for control group*]) (Figures 1, 2, 3c). There was one instance of an average control value being zero (BTBR group spots 3–4 cm^2^) and in this instance, fold‐ differences were set as zero because just two of seven *Lep^ob^* ^/^ *^ob^* mice had spots of this size and each were limited to a single spot. Fold‐differences are color coded based on the magnitude difference between the experimental groups and their controls: red ≤0.01, white =0, black ≥10. VSA endpoint fold‐differences between experimental groups and controls were scaled and zero centered. Principal components analysis (PCA) and hierarchical clustering were used to reduce dimensionality of VSA endpoint fold differences and evaluate relationships within and between experimental groups. Analysis was performed using R and Euclidean distances were used with Ward's criterion method to calculate the distance matrix and create the tree.

## 3 RESULTS AND DISCUSSION

Our first goal was to determine which VSA endpoints significantly differ between mice with three different patterns of voiding dysfunction and their respective controls.

**FIGURE 4:**
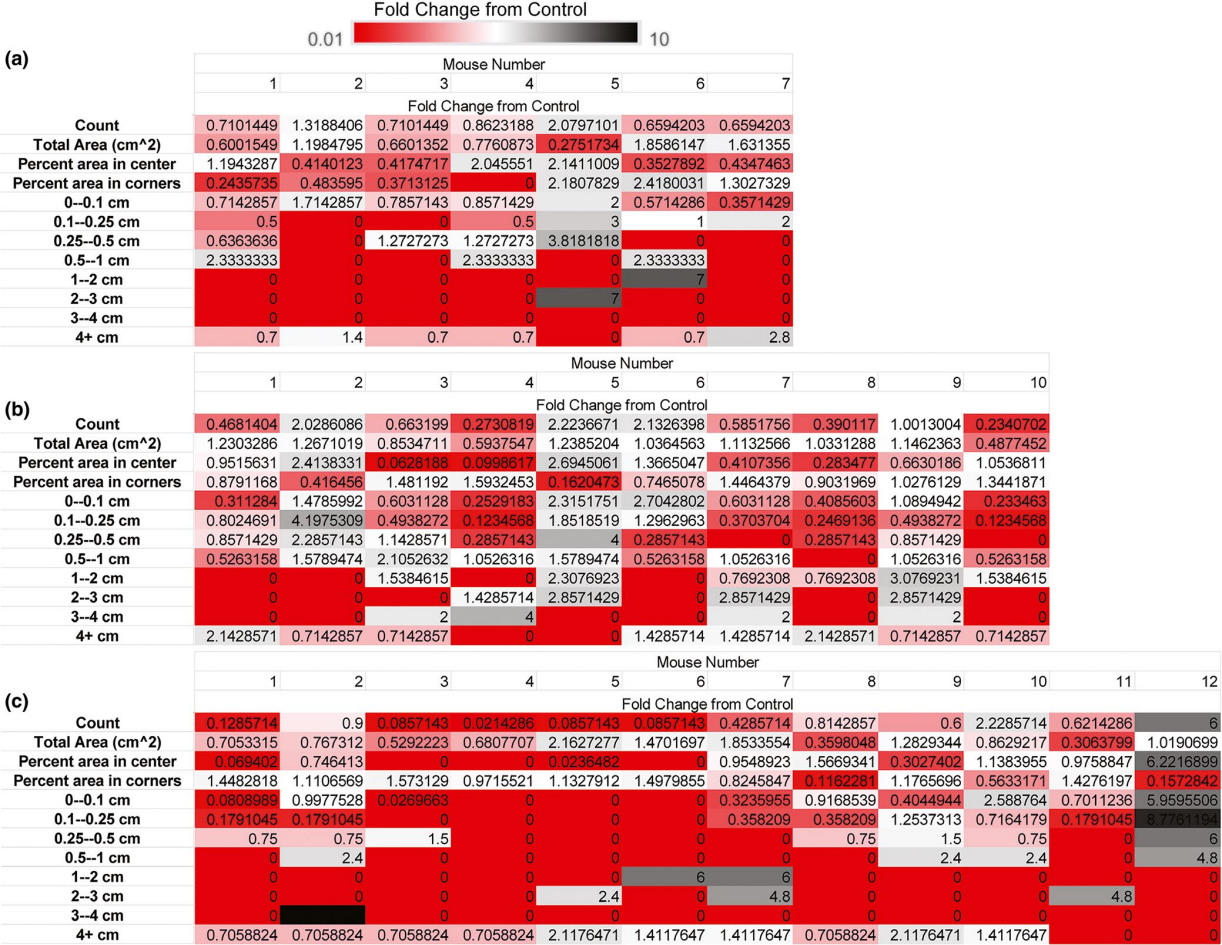
Dispersion of void spot assay endpoints in the control populations. The average control value was determined for each VWO parameter in each group and then fold‐change determined for each mouse in the control group (*fold change* = [*VSA endpoint value for each individual control mouse*]/[*average VSA endpoint value for control group*]) to evaluate dispersion of the void spot assay endpoints within the control population. (a) Diabetic diuresis control group. (b) Irritative dysfunction control group. (c) Obstructive dysfunction control group. Fold‐changes were color coded using a 3‐color scale: red ≤ 0.01, white = 0, black ≥ 10

BTBR. Cg‐*Lep* ^ob^ /WiscJ carry a spontaneous mutation in the leptin gene (*Lep^ob^)* that interferes with satiety and leads to severe obesity, and diabetes (Hudkins et al., 2010; Stoehr et al., 2000, 2004). We have confirmed that *Lep^ob^* ^/^ *^ob^* mice have elevated blood glucose and polyuria compared to *Lep* ^+/+^ mice (controls; Abler et al., 2021). We obtained VSA endpoint data from a previous study (Wegner et al., 2018). We divided VSA endpoints for each *Lep^ob^* ^/^ *^ob^* mouse by the average value of endpoints for the wild‐type controls to identify fold‐differences. VSA endpoints that differ between *Lep^ob^* ^/^ *^ob^* mice and their respective controls are spot count (*p* = 0.0041, Figure 1b), void area (*p* < 0.0001, Figure 1b), urination in the center of the paper (*p* = 0.0266, Figure 1b), small spots (0–0.1 cm^2^, *p* = 0.0123 and 0.1–0.25 cm^2^, *p* = 0.0011, Figure 1b), and large spots (4+ cm^2^, *p* = 0.0012, Figure 1b). The changes are consistent with polyuria. A heat map demonstrates the magnitude of fold‐differences in VSA endpoints between *Lep^ob^* ^/^ *^ob^* mice and controls (Figure 1c). An additional heat map shows fold‐differences in VSA endpoints between control mice and the average control value to portray dispersion in the control population (Figure 4a).

We recently characterized a model of male mouse UTI involving intraurethral administration of *E*. *coli* UTI89 and obtained VSA endpoint data from that study (Ruetten et al., 2021). The infected mouse group shown here includes only those mice that had more than 10,000 colony forming units of *E*. *coli* UTI89 in free catch urine 24 h after infection, as this group of mice is most likely to have prostate inflammation (Ruetten et al., 2021). Infected mice have a greater density of prostate‐localized inflammatory cells (CD45 immunopositive) than controls at 7 days post‐infection, the same day that VSAs were performed (*p* = 0.0357, Figure 2a). The VSA endpoints that differ between *E*. *coli* UTI89 infected mice and controls are total spot count (*p* = 0.0052, Figure 2b), number of small spots (0–0.1 cm^2^, *p* = 0.0141; 0.1–0.25 cm^2^, *p* = 0.0182; 0.25–0.5 cm^2^, *p* = 0.0365, Figure 2b), and number of large spots (4+ cm^2^, *p* = 0.0246, Figure 2b). A heat map demonstrates the magnitude of fold‐differences in VSA endpoints between infected mice and controls (Figure 2c). An additional heat map demonstrating the magnitude of fold‐differences in VSA endpoints between individual control mice and average control values is included to give an idea of the dispersion of the data in the control population (Figure 4b). It is important to note that prostatic inflammation can elicit both a frequent voiding phenotype and a urinary retention phenotype in men and mice. As discussed in the previous publication from which this data was taken, the urinary phenotype in mice is bacteria type and strain, mouse background strain, and timepoint dependent. Seven days post‐infection *E*. *coli* UTI89 produces a urinary retention phenotype in male C57BL/6J mice.

Bladder outlet obstruction is common in aging men. Adult male mice given subcutaneous, slow release implants of testosterone and estradiol (T+E2) develop anatomical and physiological changes consistent with bladder outlet obstruction: prostate enlargement, urinary retention, bladder hypertrophy, and increased risk of hydronephrosis (Nicholson et al., 2012). We recently determined that these anatomical and physiological actions of T+E2 treatment in mice derive in part from the action of estrogens (Wegner et al., 2020) and we obtained VSA endpoint data for use in the current study. We confirm that bladders from T+E2‐treated mice and filled with urine and have a greater volume than sham operated controls 8 weeks after T+E2 implantation surgery (*p* < 0.00001, Figure3a). VSA endpoints that differ between T+E2‐treated mice and their controls at 8 weeks post‐implantation surgery are spot count (*p* = 0.0097, Figure 3b), number of spots in the center of the paper (*p* = 0.0066, Figure 3b), number of spots in the corners of the paper (*p* = 0.0052, Figure 3b), and the number of small spots (0–0.1 cm^2^, *p* = 0.0040, Figure 3b). A heat map demonstrates the magnitude of fold‐differences in VSA endpoints between infected mice and controls (Figure 3c). An additional heat map demonstrates the magnitude of fold‐differences in VSA endpoints between control mice and average control values to portray dispersion in the control population (Figure 4c).

**FIGURE 5:**
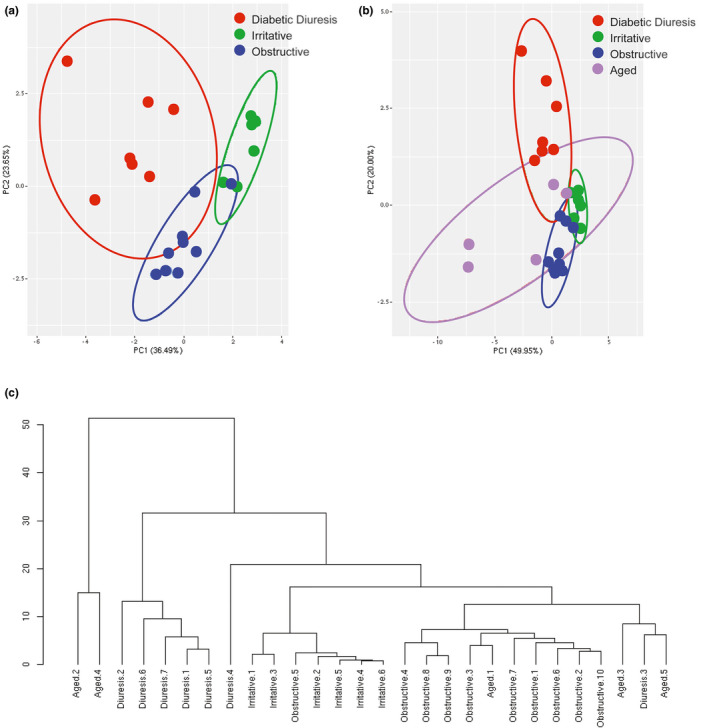
Mice with three different etiologies of urinary dysfunction uniquely cluster based on twelve VSA endpoints (the VSA phenome). The VSA phenomes of some aged mice cluster with bladder outlet obstructed mice, other aged mice cluster with diabetic mice, and other aged mouse VSA phenomes do not cluster with diabetic, obstructed, or infected mice. Ellipses depict 95% confidence interval. (a) VSA endpoints from *n* = 6–10 diabetic, urinary tract infected or bladder outlet obstructed mice, normalized to their respective controls (Figures 1c, 2c and 3c) were zero centered, scaled, and considered as equally weighted variables for Principal Components Analysis (PCA). PC1 explained 36.49% of the variance, PC2 23.65% of the variance, and the first three PCs explain 73.99% of the total variance. (b) VSA endpoints from *n* = 5 aged (96 wk) male mice were normalized to those of *n* = 5 young (8 wk) mice, zero centered, and scaled, and combined with VSA phenomes from diabetic, urinary tract infected, or bladder outlet obstructed mice for PCA. PC1 explained 49.95% of the variance, PC2 20.00% of the variance, and the first 3 PCs explain 80.25% of the total variance. (c) Hierarchical clustering was performed on the data used for PCA analysis

Results from Figures 1c, 2c, and 3c (VSA endpoints for each mouse divided by the average endpoints of controls) were used to compare the VSA phenomes of *Lep^ob^* ^/^ *^ob^* mice, *E*. *coli* infected mice, and T+E2 treated mice. VSA endpoint data for each model was zero centered and scaled and all endpoint data were equally weighted for use in PCA. The VSA phenomes from each experimental group formed fairly distinct clusters; in hierarchal clustering only one obstructive mouse grouped with the irritative group rather than the rest of its phenotype (Figure 5c). This shows for the first time that the VSA patterns of mice with voiding dysfunction are not only different from their controls but that different patterns of voiding dysfunction are distinguishable by VSA.

Aging increases the risk for a variety of disease processes that cause voiding dysfunction, including diabetes, spontaneous UTI, bladder outlet obstruction, and others. While it had previously been speculated that humans and dogs are the only species that are at risk for spontaneous prostate related voiding dysfunction, a recent study demonstrates that the mouse prostate grows spontaneously with age and that aging male mice develop urinary voiding dysfunction (Liu et al., 2019). We obtained VSA endpoint data from the previous study to test whether aging is associated with a unique VSA phenome. Surprisingly, the VSA aging phenome is not unique (Figure 5b). Instead, the VSA phenome from some aging mice formed a unique hierarchal cluster that was different from that of mice with diabetic diuresis, UTI, and bladder outlet obstruction. One aged mouse clustered with the bladder outlet obstructed mice, and other aging mice clustered with the diabetic diuresis mice (Figure 5c). These outcomes support the idea that aged mice, like men, spontaneously develop urinary dysfunction patterns representing a range of etiologies.

**FIGURE 6:**
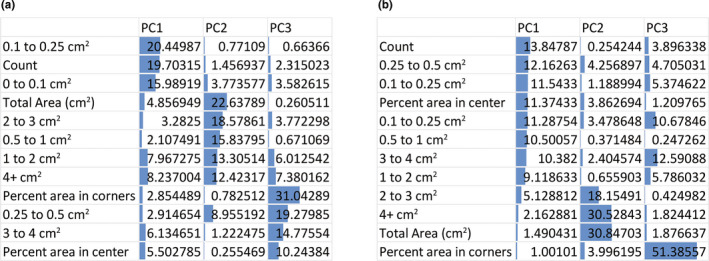
All VSA parameters contribute to successful clustering of voiding phenotypes by experimental group. Principle components (PCs) are new variables constructed during PCA that represent a direction in the data that explains a maximal amount of variance. PCs are not based on a single variable included in analysis but rather based on the contribution of several variables to form a new variable. The first three PCs generally contribute the majority of the variance. In our PCA plots (Figure 4a,b) we plot PC1 and PC2. (a) Contributions of VSA endpoints to first three PCs from *n* = 6–10 diabetic, urinary tract infected or bladder outlet obstructed mice, normalized to their respective controls. PC1 explained 36.49% of the variance, PC2 23.65% of the variance, and the first three PCs explain 73.99% of the total variance. (b) Contributions of VSA endpoints to first three PCs from *n* = 5 aged (96 wk) male mice normalized to those of *n* = 5 young (8 wk) mice and combined with VSA phenomes from diabetic, urinary tract infected, or bladder outlet obstructed mice for PCA. PC1 explained 49.95% of the variance, PC2 20.00% of the variance, and the first 3 PCs explain 80.25% of the total variance

Post‐PCA analysis, we determined the contributions of each VSA parameter to the first 3 PCs in each analysis (Figure 6). Principal components (PCs) are new variables constructed during PCA that represent a direction in the data that explains a maximal amount of variance. PCs are not based on a single variable included in analysis but rather based on the contribution of several variables to form a new variable. The first three PCs generally contribute the majority of the variance. In our PCA plots (Figure 5a,b) we plot PC1 and PC2. In our analysis of the three groups of urinary dysfunction (Figure 5a), total spot count, and number of 0–0.1 and 0.1–0.25 cm^2^ spots contributed 56% of PC1, total Area, number of 0.5–1, 1–2, 2–3, and >4 cm^2^ spots contributed 83% of PC2, and percent area in center, percent area in corner, 0.25–0.5 and 3–4 cm^2^ spots contributed 75% of PC3 (Figure 6a). In our analysis including aged mice (Figure 5b), total spot count, number of 0–0.1, 0.1–0.25, 0.25–0.5, 0.5–1, 3–4 cm^2^ spots, and percent area in the center contributed 81% of PC1, total Area, number of 2–3, and >4 cm^2^ spots contributed 80% of PC2, and percent area in corner, and 0.1–0.25 and 3–4 cm^2^ spots contributed 75% of PC3 (Figure 6b). In both analyses, each VSA parameter included contributed at least 10% of one of the first three PCs in each analysis. Based on this we recommend continuing to use all VSA parameters going forward as they all contribute to differentiating urinary phenotypes.

## 4 CONCLUSIONS

In animal studies, we perform physiologic tests to detect voiding dysfunction. Previous studies have assessed VSA variables in isolation but here we have taken an approach akin to RNA microarray analysis, using clustering approaches to reduce data dimensionality and determine if urinary physiology data can be used collectively to distinguish phenotypes of voiding dysfunction. This approach is unique because it does not assume specific meaning for any VSA endpoint, but instead considers all endpoints as equally weighted variables that contribute to a VSA phenome. Our findings support continued use of the void spot assay for etiologic and therapeutic testing in mice and demonstrates for the first time that the void spot assay is not only sensitive enough to detect differences from controls but also separate different types of voiding dysfunction. Our findings also suggest that aged mice develop urinary dysfunction consistent with the range of etiologies in men and supports their use as an ideal animal model in LUTS research. This work opens the door to future studies combining VSA data with other anatomical and physiological endpoints, increasing the power of future multivariate analysis, and facilitating model building for diagnostic urinary physiology assays in mice. As additional variables are added, additional mouse models can be incorporated into the dataset increasing accuracy and coverage. This is particularly important for mice with urinary tract infection and/or inflammation which can yield a spectrum of voiding disorders. Continued work of this nature is necessary to continue to improve the predictability of preclinical testing using mouse models.

## AUTHOR CONTRIBUTION

H.R. and C.M.V. conceived and designed the research; H.R. and T.L. performed the experiments; H.R. and G.H.H. analyzed the data; H.R., H.M.S., and C.M.V. interpreted the results of experiments; H.R. prepared the figures; H.R. and C.M.V. drafted the manuscript; H.R., G.H.H., T.T.L., H.M.S., W.A.R., D.W.S., and C.M.V. edited and revised the manuscript; H.R., G.H.H., T.T.L., H.M.S., W.A.R., D.W.S., and C.M.V. approved the final version of manuscript.
